# CA10 and CA11 negatively regulate neuronal activity‐dependent growth of gliomas

**DOI:** 10.1002/1878-0261.12445

**Published:** 2019-03-20

**Authors:** Bangbao Tao, Yiqun Ling, Youyou Zhang, Shu Li, Ping Zhou, Xiaoqiang Wang, Bin Li, Zhong Jun, Wenchuan Zhang, Chunyan Xu, Juanhong Shi, Lifeng Wang, Wenhao Zhang, Shiting Li

**Affiliations:** ^1^ Department of Neurosurgery Xinhua Hospital Shanghai Jiaotong University School of Medicine China; ^2^ Department of Nutrition Fudan University Shanghai Cancer Center China; ^3^ Department of Endocrinology The First Hospital of Taizhou Wenzhou Medical University Taizhou China; ^4^ Department of Pathophysiology Wannan Medical College China; ^5^ Department of Pathology Xinhua Hospital Shanghai Jiaotong University School of Medicine China; ^6^ Department of Hematology Xinhua Hospital Shanghai Jiaotong University School of Medicine China

**Keywords:** CA10, CA11, gliomas, microenvironment, neuroligin‐3, neurons

## Abstract

Recent studies have revealed that neurons can promote glioma growth through activity‐dependent secretion of neurotrophins, especially neuroligin‐3. It has therefore been suggested that blocking neuron‐derived neurotrophins may serve as a therapeutic intervention for gliomas. Carbonic anhydrase‐related proteins 11 and 10 (CA11 and CA10) are secreted synaptic proteins which function as neurexin ligands, and the gene‐encoding CA11 is part of a gene signature associated with radiotherapy and prognosis in gliomas. We therefore hypothesized that CA11/CA10 might participate in the neuronal activity‐dependent regulation of glioma growth. In this study, we report that CA11 secreted by depolarized cultured neurons within conditioned medium (CM) inhibited the growth of glioma cell lines. CM from depolarized neurons inhibited CA11 expression in glioma cell lines via the Akt signaling pathway. Consistently, CA11 expression was also reduced in clinical glioma samples and negatively associated with high histological grade. Low CA11 expression of gliomas was associated with short survival in four independent datasets [repository of brain neoplasia data (REMBRANDT), The Cancer Genome Atlas (TCGA) lower grade glioma (LGG), GSE4271, and GSE42669]. CA11 knockdown promoted cell growth, clone formation, and migration; inhibited apoptosis; and increased tumor size in xenografted nude mice. Similarly, CA10 and CA10 secreted by depolarized cultured neurons also inhibited the growth of glioma cell lines. Low CA10 expression was associated with short survival in REMBRANDT, TCGA LGG, and GEO GSE4271 datasets. Our results suggest that CA11 and CA10 negatively regulate neuronal activity‐dependent glioma growth and inhibit glioma aggression. Thus, CA11/CA10 may represent a potential therapeutic target for the treatment of gliomas.

**Abbreviations**
BDNF: brain‐derived neurotrophic factor
CA: carbonic anhydrase‐related proteins
CM: conditioned medium
GBM: glioblastomas
HR: hazard ratio
LGG: lower grade glioma
MOI: multiplicity of infection
REMBRANDT: repository of brain neoplasia data
shRNA: short hairpin RNA
TCGA: The Cancer Genome Atlas

## 1 Introduction

Gliomas are the most common primary brain tumors in adults (Wen and Kesari, 2008) and are among the most lethal cancers in the world, as the prognosis is very poor for patients with high‐grade gliomas (Buckner, 2003). However, the underlying molecular mechanism of its pathogenesis is far from clear. Major efforts in past studies have focused on glioma cells themselves with little attention paid to the surrounding neurons. Although interaction between tumor cells and their surrounding microenvironment has long been a topic for many peripheral tumors (Liotta and Kohn, 2001), the potential interaction between neurons and gliomas, and its contribution to the pathogenesis of gliomas, remain largely unknown. A recent study reveals that neurons could promote glioma growth through activity‐dependent secretion of neurotrophins, especially neuroligin‐3 (Venkatesh *et al*., 2015). In addition, blocking neuroligin‐3 secretion by ADAM metallopeptidase domain 10 inhibitor shows a strong inhibition on the *in vivo* growth of gliomas (Venkatesh *et al*., 2017). These studies highlight a crucial role of neuronal regulation on the aggression of gliomas and suggest that neuron‐derived neurotrophins are potential therapeutic targets for the treatment of gliomas (Johung and Monje, 2017).

However, neurotrophins such as neuroligins and brain‐derived neurotrophic factor (BDNF) are indispensable for normal cognition and memory (Huang and Reichardt, 2001). It would be difficult to choose the therapeutic target with the optimal balance between anti‐glioma efficacy and unwanted side effects before we fully understand how diverse neurotrophins are involved in this neuronal regulation of glioma behaviors. On this occasion, it is unclear whether any neuron‐derived neurotrophins could reduce glioma growth. Carbonic anhydrase‐related protein 11 (CA11) is a secreted protein mainly expressed in the brain. It belongs to the well‐conserved carbonic anhydrase‐related proteins, which include CA8, CA10, and CA11 (Aspatwar *et al*., 2010). Their function is almost unknown as they have lost catalytic activity. In spite of this, several studies suggest that CA8 appears to play important roles in several types of cancer (Ishihara *et al*., 2006; Miyaji *et al*., 2003; Nishikata *et al*., 2007) and CA11 is a gene signature associated with radiotherapy and prognosis in gliomas (Li *et al*., 2017). More interestingly, a recent study suggests that CA10 and CA11 are neurexin ligands, just like neuroligin‐3 (Sterky *et al*., 2017). Neurexin is a presynaptic transmembrane protein, and its extracellular domain interacts with proteins such as neuroligin‐3 in the synaptic cleft to form and modulate synapses (Sudhof, 2008). Given that CA11 is potentially involved in oncogenesis and that CA11/CA10 function as neurexin ligands the same as neuroligin‐3, we hypothesize that CA11/CA10 might participate in the neuronal activity‐dependent regulation of glioma growth.

In this study, we report that depolarized cultured neurons secreted CA10 and CA11 within the conditioned medium (CM) inhibited the growth of glioma cell lines. CM from depolarized neurons inhibited CA11 expression in glioma cell lines via the Akt signaling pathway. Consistently, CA11 expression was also reduced in clinical glioma samples and negatively associated with high histological grade. Low CA11 expression of gliomas was associated with short survival in four independent datasets. In addition, CA11 knockdown promoted aggressive tumor behaviors in various *in vitro* and *in vivo* assays. Similarly, depolarized cultured neurons also secreted CA10, and CA10 inhibited the growth of glioma cell lines. Low CA10 expression was associated with short survival in three datasets. Our results support CA11 and CA10 sharing a conserved function in gliomas by negatively regulating neuronal activity‐dependent glioma growth and inhibiting glioma aggression. Thus, CA11/CA10 may represent a potential therapeutic target for the treatment of gliomas.

## 2 Materials and methods

### 2.1 Clinical samples

The study was approved by the Review Boards of Xinhua Hospital (Shanghai, China) and conducted according to the principles of the Declaration of Helsinki. Written informed consent was obtained from each patient. Thirty‐five primary glioma tissue samples were collected in Xinhua Hospital. Seven normal brain tissue samples were obtained from nonglioma patients undergoing brain surgery. All cases were confirmed by pathological diagnosis. Grading of gliomas was performed according to the 2007 World Health Organization Classification criteria. In this study, all cases of gliomas were classified as low grade (WHO I and II, *n* = 14) or high grade (WHO III and IV, *n* = 21) for statistical analysis. Tissues were freshly resected during surgery, immediately stored in liquid nitrogen for subsequent extraction of protein and RNA, or prepared as paraffin‐embedded blocks for immunochemistry analysis.

### 2.2 Cell cultures

Glioma cell lines U87 and U251 from ATCC were maintained in Dulbecco's modified essential medium (DMEM) with 10% FBS, 100 U·mL^−1^ penicillin, and 100 mg·mL^−1^ streptomycin. HEK293T from the American Type Culture Collection (ATCC) was maintained in DMEM medium with 10% FBS, 100 U·mL^−1^ penicillin, and 100 mg·mL^−1^ streptomycin. Cells were cultured in a humidified atmosphere with 5% CO_2_ at 37 °C. Cell line authentication was performed to avoid cross‐contamination.

Rat primary cortical neurons were isolated from embryonic day 17 Sprague‐Dawley rat brains. Briefly, rat cerebral cortices were isolated in cold DMEM and digested for 6 min at 37 °C in 0.25% trypsin followed by adding DMEM with 10% FBS to terminate digestion. After filtered through a 70‐μm cell strainer, cells were centrifuged at 800 ***g*** for 3 min and resuspended in DMEM with 10% FBS. Cells were plated in poly‐d‐lysine‐coated dishes and further cultured in Neurobasal supplemented with B27, 0.5 mm glutamax and 5‐fluorouracil for 10–12 days.

### 2.3 Preparation of conditioned medium

To induce depolarization of primary rat neurons, they were incubated with medium containing 50 mm KCl for 2 h at day *in vitro* 10. The medium was then replaced by normal medium and neurons were further cultured for 48 h before the collection of CM. For HEK293T cells, cells were transfected with CA11 over‐expressing plasmid for 24 h and the medium was replaced by FBS‐free medium for 48 h. The CM was collected by centrifuge for 5 min at 1000 ***g***. CM was concentrated using Amicon® Ultra 15 mL Centrifugal Filters (10 kD) (MilliporeSigma, Temecula, CA, USA).

### 2.4 CA11 over‐expression and knockdown

The coding sequences of human CA11 (NM_001217.4) and CA10 (NM_001082533.1) were cloned into pcDNA3.1‐B^−^ plasmid, respectively. Plasmids were transfected using Lipofectamine 2000 (Invitrogen, Carlsbad, CA, USA). To perform CA11 knockdown, the short hairpin RNA (shRNA) targeting human CA11 mRNA sequence (forward: TGGCCAGTACCTCTAACCCATTTCAAGAGAATG GGTTAGAGGTACTGGCCTTTTTTC; reverse: TCGAGAAAAAAGATGGGTTAGAGGTACT GGCTCTCTTGAAGCCAGTACCT CTAACCCATCA) and its scrambled shRNA were constructed into the pLentiLox3.7 (pLL3.7) lentiviral vector. Target sequences were underlined. The lentivirus was packaged and amplified in HEK293T cells. Cell lines were infected at a multiplicity of infection (MOI) of 5.

### 2.5 Cell proliferation assay

Cell lines were seeded into a 96‐well plate in triplicate at the concentration of 4 × 10^3^ cells per well. The cell growth was measured by 3‐(4,5‐dimethylthiazol‐2‐yl)‐2,5‐diphenyltetrazolium bromide (MTT) assay at indicated times. Cells were incubated with 5 mg·mL^−1^ MTT for 4 h and subsequently solubilized in DMSO. The absorbance at 570 nm was then measured using an ELISA reader. The MTT assay experiments were repeated for three times.

### 2.6 Clone formation assay

Cells lines infected with CA11 shRNA were plated at the final concentration of 5 × 10^4^/L in duplicate in a six‐well plate. After incubation at 37 °C for 14 days, cells were washed with PBS, fixed with 4% paraformaldehyde, and stained with crystal violet solution in methanol for 15 min. The experiments were repeated for three times.

### 2.7 *In vitro* cell migration assay

Cell lines infected with CA11 shRNA were trypsinized and resuspended as single‐cell suspension. A total of 1 × 10^5^ cells in 0.2 mL serum‐free DMEM were seeded in 8‐μm pore chambers inserted in a Transwell apparatus (Corning, Corning, NY, USA). Then, 600 μL DMEM with 10% FBS was added to the lower chamber. After incubation for 24 h at 37 °C, the cells on the top surface of the insert were removed and the cells that migrated to the bottom surface of the insert were fixed in 100% methanol and stained with 0.5% crystal violet. The experiments were repeated three times.

### 2.8 Apoptosis assay

Cells lines were infected with CA11 shRNA for 2 days. Cells were trypsinized, washed, and stained with Annexin V‐PE Apoptosis kit (Abcam, ab14155, Cambridge, MA, USA) in the dark for 15 min at room temperature. The stained cells were then analyzed by MoFlo XDP (Beckman Coulter, Inc, Miami, FL, USA). The apoptosis assay experiments were repeated three times.

### 2.9 *In vivo* xenografted model

The animal experiments were conducted in accordance with the animal welfare guidelines of Xinhua Hospital. Female athymic nude mice (6 weeks old, BALB‐c/nu/nu strain) were kept in specific pathogen‐free conditions and were randomly assigned to two groups: CA11 shRNA and scramble groups, five animals per group. U251 cells were infected with CA11 shRNA or scramble lentivirus at MOI of 10. A total of 1 × 10^6^ cells were implanted into subcutaneous tissue of a posterior limb with a 26‐gauge needle/1 mL syringe. Tumor size was measured after 4 weeks by measuring the length (*L*) and width (*W*) of xenografted tumors with a Vernier caliper. Tumor size was calculated as follows: *L* × (*W*)2/2.

### 2.10 Quantitative real‐time PCR

To detect the relative CA11 mRNA level, quantitative real‐time PCR (qPCR) was performed. Briefly, total RNA was extracted from tissues or cell lines using TRIzol reagent, according to the manufacturer's protocol. The cDNA was generated with 1 μg total RNA using MMLV reverse transcriptase (Promega, Madison, WI, USA) and random primers. Actin was used as an endogenous control. The qRT‐PCR primers were as follows: CA11: TACAGCCACCGACTCAGTGAA (forward) and GGTTGAAGTGAATGAGCTGCAC (reverse); actin: ACCAACTGGGACGACATGGAGAAA (forward) and TAGCACAGCCTGGATAGC AACGTA (reverse). The fold change for each target gene relative to the control group was calculated using the ΔΔ*C*_t_ method (Schmittgen and Livak, 2008).

### 2.11 Western blot

Proteins were extracted from tissues or cell lines using RIPA lysis buffer and denatured with 5 × SDS loading buffer. CM was concentrated using Amicon® Ultra 15‐mL Centrifugal Filters (10 kD) before boiling with 5 × SDS loading buffer. Protein samples were resolved by SDS/PAGE and probed with the following antibodies. CA11, CA10, BDNF, Neuroligin‐3, HA, Akt, and p‐Akt (Ser473) antibodies were from Sigma‐Aldrich (HPA041778), Sigma‐Aldrich (HPA057837; Madison, WI, USA), Abcam (ab203573), Abcam (ab192880), Sigma‐Aldrich (H6908), CST (Boston, MA, USA; 4691), and CST (4060), respectively.

### 2.12 Luciferase assay of CA11 promoter activity

The 500‐bp promoter of the human CA11 was amplified by PCR and cloned into pGL4.10 vector. For luciferase reporter assays, cells were transiently transfected with pGL4.10 containing CA11 promoter using Lipofectamine 2000 for 24 h and treated with CM for indicated times. Reporter gene activity was measured by the dual‐luciferase assay system (Promega). Renilla luciferase activity was used to normalize for transfection efficiency. The data were presented as fold change relative to the control group. The luciferase assay experiments were repeated three times.

### 2.13 Immunochemistry

Serial sections of paraffin‐embedded tissues at the thickness of 6 μm were prepared. Immunostaining of the sections with CA11 antibody (1 : 100 dilution) was performed according to the instruction of VectaStain Universal ABC kit (Vector Laboratories, Burlingame, CA, USA). The slides were counterstained with hematoxylin. The staining was analyzed by the Quickscore method (Detre *et al*., 1995). Briefly, each case was represented by five random visual fields. The number of positive cells was counted, and the positive rate (*R*) was calculated by the total number of positive cells divided by the total number of all cells in five fields. The positive rate was further scored as: 1, < 25%; 2, < 50%; 3, > 50%. The staining intensity (*I*) was scored as 1 for weak, 2 for moderate, and 3 for strong. The Quickscore equals *R* × *I*. The Quickscore of each case was calculated as the average Quickscore of five fields. The analysis of the immunochemistry results was performed double‐blind.

### 2.14 Survival analysis of open‐access datasets

As a repository of brain neoplasia data (REMBRANDT) gliomas, expression data and clinical data were downloaded from REMBRANDT website (http://www.betastasis.com/glioma/rembrandt/kaplan_meier_survival_curve/). For The Cancer Genome Atlas (TCGA) lower grade glioma (LGG) and TCGA glioblastomas (GBM), expression data and clinical data were downloaded from cBioPortal for Cancer Genomics (http://www.cbioportal.org/). For GSE4271 and GSE42669, expression data and clinical data were downloaded from GEO database (https://www.ncbi.nlm.nih.gov/geo/). In each dataset, the patients with CA11 expression higher than the median level were classified as the CA11‐high expression group, while the patients with CA11 expression lower than the median level were classified as the CA11‐low expression group. The Kaplan–Meier method was used to create survival curves, and the log‐rank test was used to compare survival curves of CA11‐high and CA11‐low expression groups.

### 2.15 Statistical analysis

Statistical analysis was performed using graphpad prism software (GraphPad Software, Inc., San Diego, CA, USA). For data from cell lines, all data were presented as mean ± SD and statistical analysis was performed by the two‐tailed Student's *t*‐test for two groups and one‐way ANOVA with Newman–Keuls *post hoc* test for more than two groups. For data from clinical samples, all data were presented as whiskers‐box plots. The nonparametric Mann–Whitney *U* test was used for two groups and Kruskal–Wallis test followed by *post hoc* Dunn's multiple comparison test was used for more than two groups. Statistically significant differences were defined as *P* < 0.05: *P < 0.05, **P < 0.01, ***P < 0.001.

## 3 Results

### 3.1 Neuron‐secreted CA11 reduces the neuronal activity‐dependent growth in glioma cell lines

**Figure 1:**
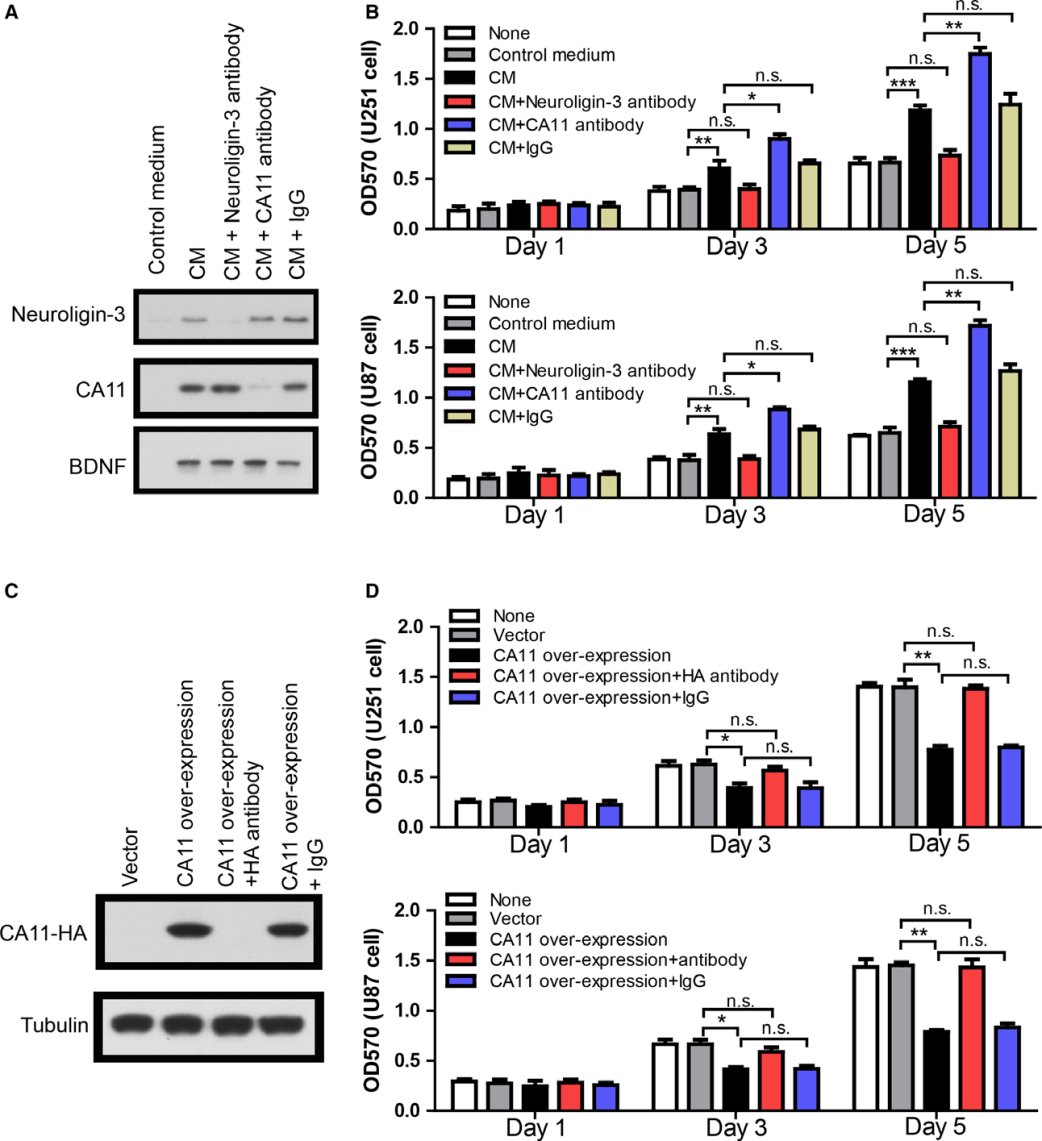
Neuron‐secreted CA11 reduces the neuronal activity‐dependent growth in glioma cell lines. (A) Representative western blot showing neuroligin‐3, CA11, and BDNF levels in the CM from control neurons and depolarized neurons with or without immunodepletion of neuroligin‐3 and CA11, respectively. (B) MTT assay results showing the growth of U251 and U87 cells treated with indicated neuronal medium at Day 1, Day 3, and Day 5, respectively (*n* = 5 biologically independent replicates). (C) Representative western blot showing CA11 levels in the CM from HEK293T cells over‐expressing HA‐tagged human CA11 with or without immunodepletion of CA11. (D) MTT assay results showing the growth of U251 and U87 cells treated with indicated HEK293T CM at Day 1, Day 3, and Day 5, respectively (*n* = 5 biologically independent replicates). *P < 0.05; **P < 0.01; ***P < 0.001 by one‐way ANOVA with Newman–Keuls *post hoc* test. The error bars in all the subfigures represent SD.

To activate cultured rat cortical neurons, we used high KCL medium to induce depolarization of neurons. The CM was collected, concentrated, and analyzed for CA11, neuroligin‐3, and BDNF levels by western blot. The result shows that, compared with control medium from nondepolarized neurons, CM from depolarized neurons contains much higher levels of CA11, neuroligin‐3, and BDNF (Fig. 1A). To determine the effects of CA11 and neuroligin‐3, they were depleted from CM using their antibodies, respectively. Western blot shows that CA11 or neuroligin‐3 was specifically eliminated by immunodepletion as BDNF level remains unchanged, and depletion using IgG did not change the levels of CA11 or neuroligin‐3 (Fig. 1A).

To evaluate the effects of CM on the growth of gliomas, we treated two glioma cell lines, U251 and U87, with CM or immunodepleted CM, and measured cell proliferation with MTT assay at Day 1, Day 3, and Day 5. MTT results show that, compared wiith control medium, CM promoted cell proliferation, and neuroligin‐3 depletion almost abolished the effects of CM (Fig. 1B). These results are consistent with a previous study reporting that CM from activated neurons promoted glioma growth largely through neuroligin‐3 (Venkatesh *et al*., 2015). Surprisingly, CA11 depletion further enhanced cell proliferation, suggesting that neuron‐secreted CA11 within CM could inhibit cell proliferation.

We notice that the effect of neuroglin‐3‐depleted CM on cell growth appears neutral, although CA11 is still present. It is likely that BDNF and GRP78 within neuronal CM may antagonize the inhibitory effect of CA11. To test the inhibitory effect of CA11 directly, we prepared CA11‐CM from HEK293T cells over‐expressing HA‐tagged human CA11. CM was concentrated and analyzed by western blot using HA antibody. The result shows that CM contained a large amount of secreted CA11, and immunodepletion with HA antibody totally eliminated CA11 from CM (Fig. 1C). U251 and U87 were treated with CM or immunodepleted CM, and cell proliferation was measured with MTT assay at Day 1, Day 3, and Day 5. The results show that CA11‐containing CM inhibited cell proliferation, and CA11 depletion abolished its inhibitory effects (Fig. 1D). These results support neuron‐secreted CA11 as a negative modulator of neuronal activity‐dependent glioma growth.

As the high homology between CA10 and CA11 (66% similarity) suggests that they may share redundant functions, we also investigated whether CA10 was secreted from the depolarized neurons. We find that CA10 was secreted into culture medium upon high KCL treatment (Fig. S1A). To test directly the effect of CA10 on glioma growth, the CM from HEK293 cell over‐expressing HA‐tagged CA10 was prepared (Fig. S1B). The effects of CA10 CM on two gliomas cell lines were measured by MTT assay, and the results show that CA10 CM had a similar inhibitory effect (Fig. S1C). Depletion with HA antibody abolished the inhibitory effect of CA10 CM.

### 3.2 Conditioned medium from depolarized neurons inhibits CA11 expression in glioma cell lines

**Figure 2:**
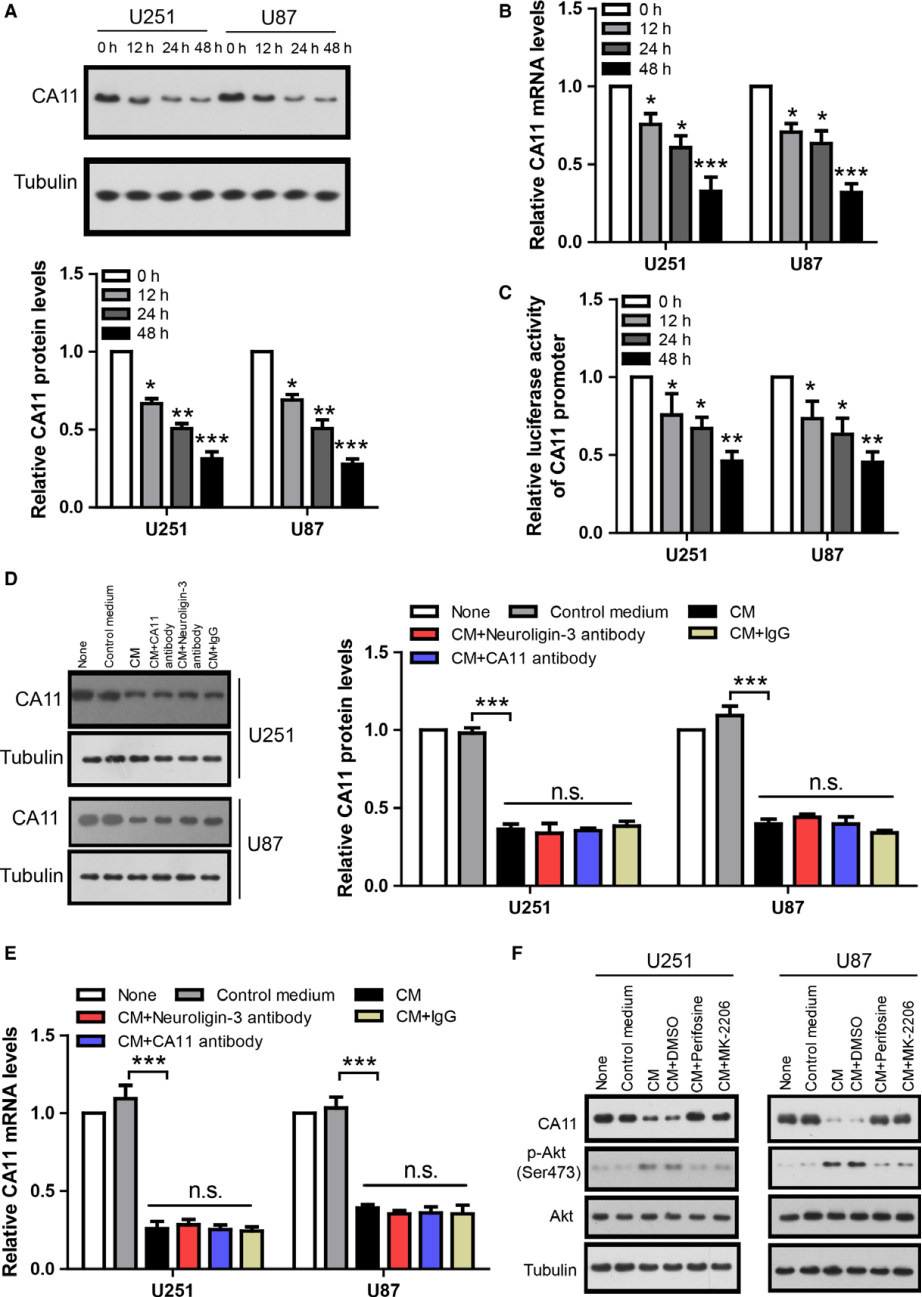
CM from depolarized neurons inhibits CA11 expression in glioma cell lines. (A) Representative western blot and its quantification showing CA11 protein levels in U251 and U87 cells treated with CM from depolarized neurons for indicated time (*n* = 4 biologically independent replicates). (B) The qRT‐PCR results showing CA11 mRNA levels in U251 and U87 cells treated with CM from depolarized neurons for indicated time (*n* = 4 biologically independent replicates). (C) Luciferase assay results showing CA11 promoter activity in U251 and U87 cells treated with CM from depolarized neurons for indicated time (*n* = 4 biologically independent replicates). (D) Representative western blot and its quantification showing CA11 protein levels in U251 and U87 cells treated with CM from depolarized neurons with or without immunodepletion of neuroligin‐3 and CA11 for 48 h (*n* = 4 biologically independent replicates). (E) The qRT‐PCR results showing CA11 mRNA levels in U251 and U87 cells treated with CM from depolarized neurons with or without immunodepletion of neuroligin‐3 and CA11 for 48 h (*n* = 4 biologically independent replicates). (F) Western blots showing the indicated proteins in U251 and U87 cells co‐treated with CM from depolarized neurons and Akt signaling inhibitors (1 μm Perifosine or 1 μm MK‐2206) for 48 h. *P < 0.05; **P < 0.01; ***P < 0.001 by one‐way ANOVA with Newman–Keuls *post hoc* test. The error bars in all the subfigures represent SD.

To investigate the effects of CM from depolarized neurons on glioma CA11 expression, U251 and U87 were treated with CM for 0, 12, 24, or 48 h, and CA11 expression was analyzed by western blot and qRT‐PCR, respectively. The results show that CM reduced the protein (Fig. 2A) and mRNA (Fig. 2B) levels of CA11 in a time‐dependent manner. Consistently, luciferase assay shows that CM also reduced CA11 promoter activity (Fig. 2C). Taken together, these results suggest CM from depolarized neurons inhibited glioma CA11 expression. Importantly, previous study also explored the transcriptome of glioma cells exposed to active CM and their RNA sequencing results show a clear trend of reduced CA11 expression under CM treatment (fold change = −0.75, *P* = 0.058, GSE62563) (Venkatesh *et al*., 2015).

To test whether neuron‐secreted neuroligin‐3 or CA11 mediated the inhibitory effect of CM on glioma CA11 expression, neuroligin‐3 and CA11 were depleted from neuronal CM. U251 and U87 were treated with CM or immunodepleted CM for 48 h, and CA11 expression was analyzed by western blot (Fig. 2D) and qRT‐PCR (Fig. 2E), respectively. The results show that neither neuroligin‐3 nor CA11 within CM had effects on glioma CA11 expression. Thus, the inhibitory effect of CM on glioma CA11 expression is mediated through other components within CM.

Although the exact components within neuronal CM which are responsible for the inhibition of glioma CA11 expression are unclear, we found that this inhibitory effect of CM is mediated through the Akt signaling pathway as co‐treatment of glioma cells with CM, and inhibitors for Akt signaling (1 μm Perifosine or 1 μm MK‐2206) for 48 h blocked the inhibitory effect (Fig. 2F).

### 3.3 CA11 expression is reduced in glioma tissues

**Figure 3:**
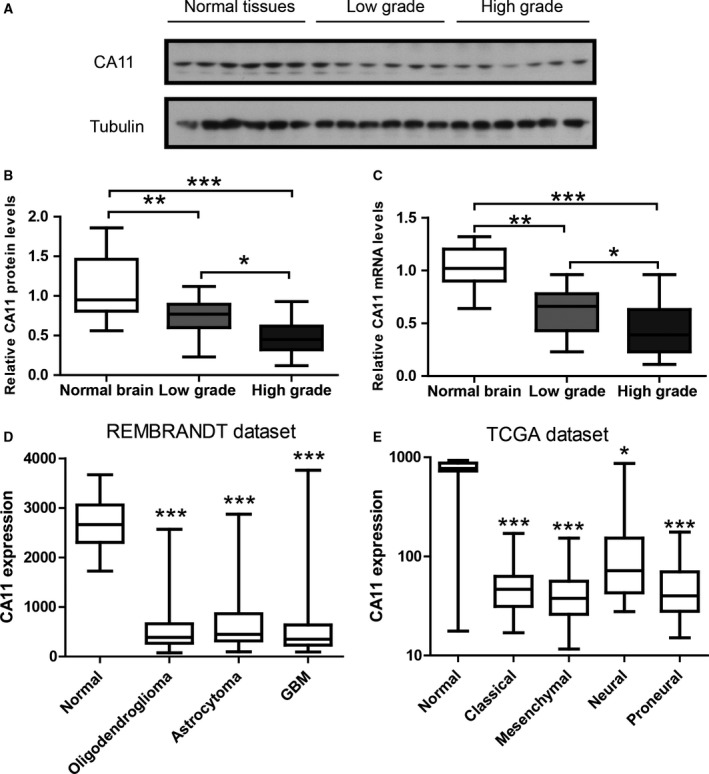
CA11 expression is reduced in gliomas. (A) Representative western blot and (B) its quantification showing CA11 protein levels in normal brain tissues (*n* = 7), low‐grade gliomas (*n* = 14), and high‐grade gliomas (*n* = 21). Data were presented as whiskers‐box plots. (C) The qRT‐PCR results showing CA11 mRNA levels in the same samples. Data were presented as whiskers‐box plots. (D) CA11 expression data in REMBRANDT glioma dataset (total *n* = 524) and (E) TCGA GBM dataset (total *n* = 454). Data were presented as whiskers‐box plots. *P < 0.05; **P < 0.01; ***P < 0.001 by Kruskal–Wallis test followed by *post hoc* Dunn's multiple comparison test.

To directly evaluate CA11 expression in primary glioma tissues, we performed western blot and qRT‐PCR in 35 cases of gliomas and seven cases of normal brain tissues. The representative image of western blot (Fig. 3A) and its quantification (Fig. 3B) shows that CA11 protein levels were reduced in gliomas compared with normal tissues, and were further reduced in high‐grade gliomas. Consistently, qRT‐PCR result shows that CA11 mRNA levels were also reduced in gliomas compared with normal tissues, and were further reduced in high‐grade gliomas (Fig. 3C). To avoid potential bias from small sample size, we sought to cross‐validate CA11 reduction in open‐access databases. Expression data from both the REMBRANDT glioma dataset (Madhavan *et al*., 2009) (total *n* = 524, Fig. 3D) and the TCGA GBM dataset (total *n* = 454, Fig. 3E) show a robust reduction of CA11 expression in gliomas compared with normal tissues. We also looked at CA10 expression in the REMBRANDT glioma dataset and TCGA GBM dataset. The data show robust downregulation of CA10 expression in tumor tissues in these two large datasets (Fig. S2).

**Figure 4:**
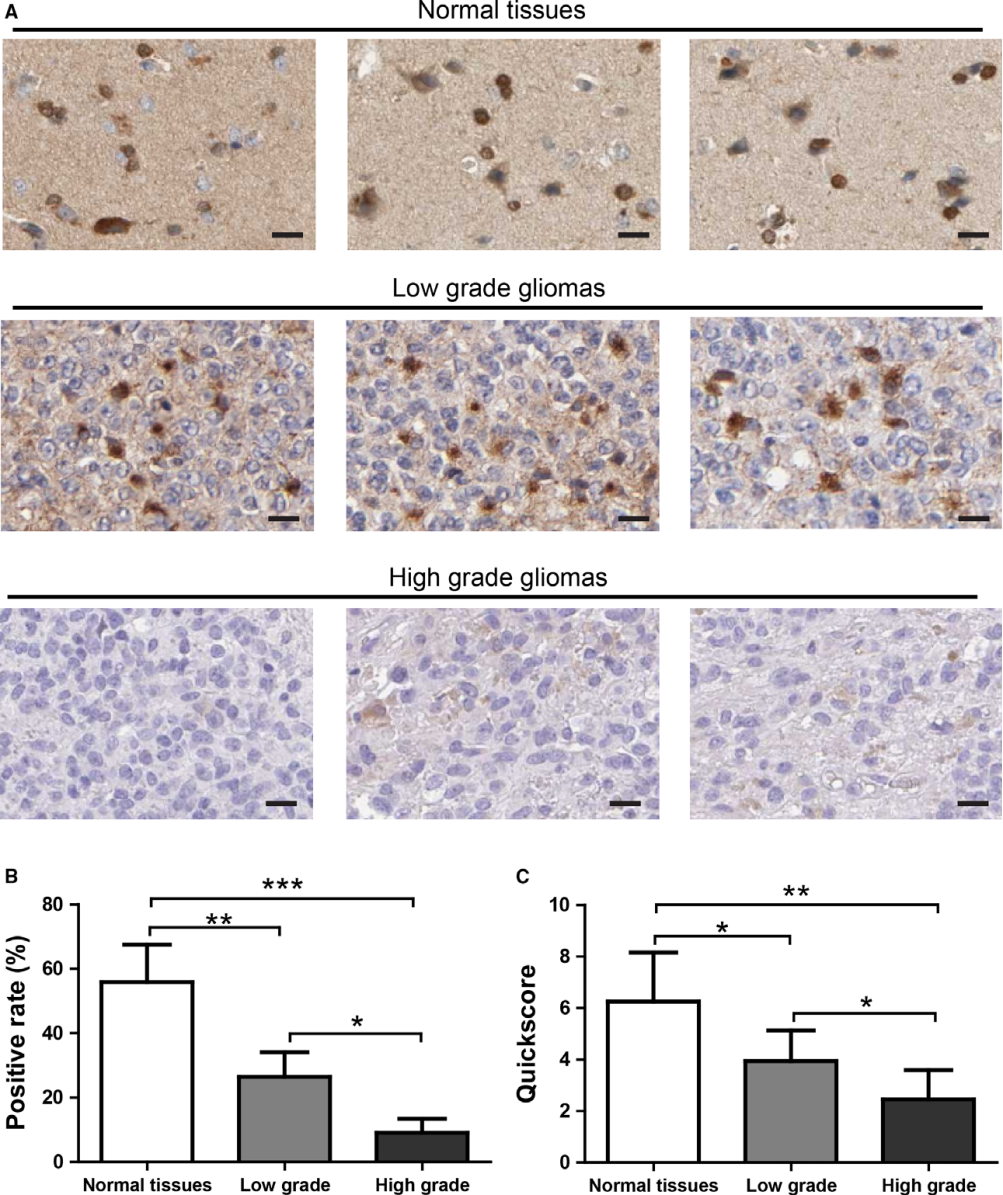
CA11 immunostaining is reduced in gliomas. (A) Representative images of CA11 staining in normal tissues (*n* = 7), low‐grade (*n* = 14), and high‐grade gliomas (*n* = 21). (B) Quantification of CA11 staining by positive rate in normal tissues, low‐grade, and high‐grade gliomas. (C) Quantification of CA11 staining by Quickscore in normal tissues, low‐grade, and high‐grade gliomas. Data were presented as mean ± SD. *P < 0.05; **P < 0.01; ***P < 0.001 by Kruskal–Wallis test followed by *post hoc* Dunn's multiple comparison test. The error bars in all the subfigures represent SD. Scale bar:  50 μm.

The reduction of CA11 expression was further confirmed by immunochemistry in the same samples. The representative images show a clear cytoplasmic staining pattern of CA11 in neurons as well as glial cells (Taniuchi *et al*., 2002). In contrast, CA11 immunostaining was almost lost in high‐grade gliomas (Fig. 4A). CA11 staining results were further analyzed by a semi‐quantitative Quickscore method. Consistently, both the positive rate (Fig. 4B) and the Quickscore (Fig. 4C) were reduced in gliomas compared with normal tissues, and were further reduced in high‐grade gliomas. These results suggest that CA11 expression was greatly reduced in gliomas compared with normal tissues, and was negatively associated with high histological grade. Thus, CA11 reduction may play a crucial role in the aggressiveness of gliomas.

### 3.4 Low CA11 expression is associated with short survival

**Figure 5:**
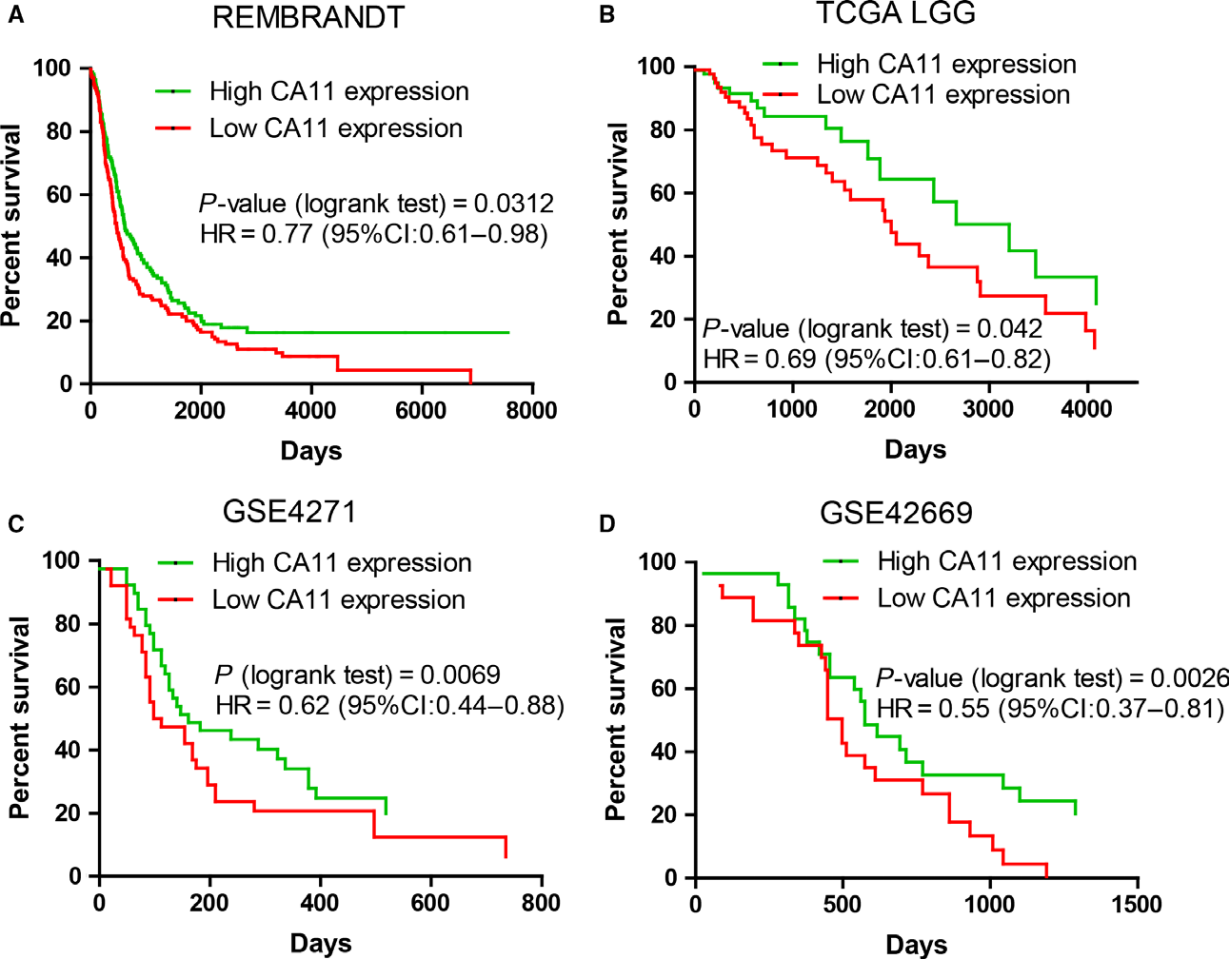
Low CA11 expression is associated with short survival. Kaplan–Meier survival curves of patients classified by CA11 expression in (A) REMBRANDT glioma dataset (*n* = 524, HR = 0.77, *P* = 0.0312), (B) TCGA LGG dataset (*n* = 532, HR = 0.69, *P* = 0.042), (C) GSE4271 (*n* = 77, HR = 0.62, *P* = 0.0069), and (D) GSE42669 (*n* = 58, HR = 0.55, *P* = 0.0026).

To assess the effect of CA11 expression on clinical prognosis, we used the Kaplan–Meier survival curve to analyze the effects of CA11 expression on the survival of patients in four independent datasets from open‐access databases: REMBRANDT gliomas dataset (*n* = 524, Fig. 5A), TCGA LGG dataset (*n* = 532, Fig. 5B), GSE4271 dataset (*n* = 77, Fig. 5C) (Phillips *et al*., 2006), and GSE42669 (*n* = 58, Fig. 5D) (Joo *et al*., 2013). In each dataset, patients were classified into CA11‐high expression and CA11‐low expression groups according to the median expression levels of CA11. The results show that patients with low CA11 expression had a significantly shorter survival time than those with high expression in REMBRANDT [hazard ratio (HR) = 0.77, *P* = 0.0312], TCGA LGG (HR = 0.69, *P* = 0.042), GSE4271 (HR = 0.62, *P* = 0.0069), and GSE42669 (HR = 0.55, *P* = 0.0026) datasets.

As many factors such as CA11 expression and histopathological grade may potentially affect the survival of patients, and there was a correlation between CA11 expression and histopathological grade, it is unclear whether CA11 expression is an independent factor influencing survival. To address this question, we used a multivariate Cox proportional hazards model to analyze the survival in the TCGA LGG dataset. The results show that both CA11 expression (*P* = 0.041) and grades (*P* = 0.03) are independent factors affecting survival (Table S1). This again supports the important role CA11 expression plays in gliomas, and that its effect on survival cannot be attributed to its correlation with histopathological grade. Thus, CA11 reduction is strongly associated with poorer prognosis in patients with gliomas.

We noticed that CA11 expression had no effect on survival in the TCGA GBM dataset (HR = 1.069, *P* = 0.57; Fig. S3). The reason for this discrepancy is not clear, but it is important to note that CA11 downregulation of glioma tissues in TCGA GBM is so robust that its expression values are generally below 100. In contrast, CA11 expression values of glioma tissues in the REMBRANDT dataset are generally above 500. When CA11 expression in the TCGA GBM dataset is very low and close to the detection threshold, it may be less accurate to classify the patients into CA11‐high and CA11‐low groups.

We also analyzed the effect of CA10 expression on survival in the same datasets (Fig. S4). The results show that, similar to CA11, low CA10 expression was associated with short survival in REMBRANDT gliomas (HR = 0.55, *P* < 0.0001), TCGA LGG (HR = 0.83, *P* = 0.003), and GSE4271 (HR = 0.79, *P* = 0.035), but not in GSE42669 (*P* = 0.66) or TCGA GBM (*P* = 0.927). Thus, the effect of CA10 on survival is reproducible in three independent datasets, and two of them (REMBRANDT gliomas and TCGA LGG) have large sample sizes. This again supports that CA10 appears to share a conserved function with CA11 in gliomas.

### 3.5 CA11 knockdown promotes aggressive behaviors of gliomas

**Figure 6:**
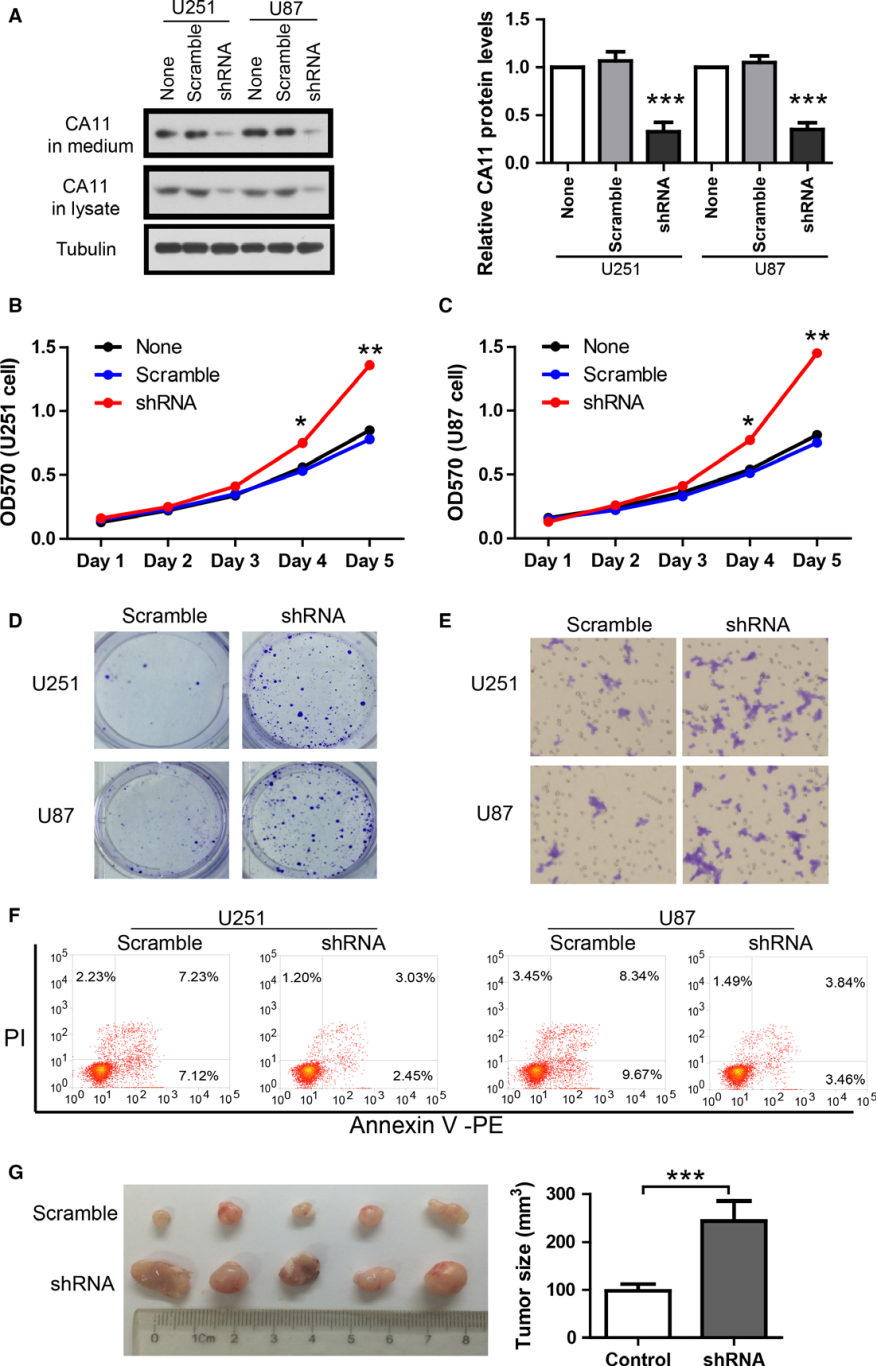
CA11 knockdown promotes aggressive tumor behaviors. (A) Representative western blot and its quantification showing the efficiency of CA11 knockdown in U251 and U87 cell lines (*n* = 3 biologically independent replicates). MTT assay showing the growth curves of U251 (B) and U87 (C) after CA11 knockdown (*n* = 4 biologically independent replicates). (D) Representative images of clone formation of U251 and U87 after CA11 knockdown. (E) Representative images of cell migration of U251 and U87 after CA11 knockdown. (F) Representative results of Annexin V‐PE apoptosis assay showing the effects of CA11 knockdown in U251 and U87 cell lines. (G) Image and quantification of tumor size in xenografted nude mice after CA11 knockdown in U251 cell line (*n* = 5 biologically independent replicates). *P < 0.05; **P < 0.01; ***P < 0.001 by two‐tailed Student's *t*‐test for two groups and one‐way ANOVA with Newman–Keuls *post hoc* test for three groups. The error bars in all the subfigures represent SD.

To explore the potential effects of reduced CA11 expression on the behaviors of gliomas, we investigated the effects of CA11 knockdown on proliferation, clone formation, *in vitro* migration, apoptosis, and *in vivo* tumor formation in glioma cell lines. U251 and U87 cells were infected with CA11 shRNA or scramble lentivirus at a MOI of 5 for 48 h. Cell lysates and culture medium were analyzed by western blot. The results show that CA11 shRNA efficiently downregulated endogenous CA11 protein levels in both cell lysates and culture medium (Fig. 6A). The effect of CA11 knockdown on cell proliferation was then measured by MTT assay and the results show that CA11 knockdown promoted cell proliferation in these two cell lines (Fig. 6B,C). In the clone formation assay, CA11 knockdown increased clone numbers (Fig. 6D). In addition, CA11 knockdown increased cell migration (Fig. 6E) in both cell lines. The effects of CA11 knockdown on apoptosis were measured by Annexin V‐PE apoptosis assay, and the results show that CA11 knockdown inhibited apoptosis (Fig. 6F). Finally, to provide *in vivo* evidence that CA11 is indeed important for tumor formation, we established subcutaneously xenografted model in nude mice. Ten nude mice were randomly assigned to CA11 shRNA group (*n* = 5) and scramble group (*n* = 5). Four weeks after implantation, mice were sacrificed and the tumor tissues were collected. The results show that the tumor size of CA11 shRNA group was significantly larger than that of scramble group (Fig. 6G). Taken together, these results suggest that CA11 reduction promotes aggressive tumor phenotypes, consistent with the fact that low CA11 expression is associated with short survival and high histological grade in patients.

## 4 Discussion

Diverse neurotrophins are essential components of the nervous system, and play important roles in cognition (Egan *et al*., 2003), emotion (Duman and Monteggia, 2006), and behavior (Chen *et al*., 2006). The potential involvement of neurotrophins in various cancers, including myeloma (Pearse *et al*., 2005), breast cancer (Vanhecke *et al*., 2011), lung cancer (Ricci *et al*., 2001), medullary thyroid carcinoma (McGregor *et al*., 1999), hepatocellular carcinoma (Yang *et al*., 2005), gastric cancer (Okugawa *et al*., 2013), and even gliomas (Birnbaum *et al*., 2007; Johnston *et al*., 2007), has also been recognized for a long time. However, the direct link between neurons and gliomas through activity‐dependent neuroligin‐3 secretion is established by a recent pioneering study by Venkatesh *et al*. (2015). The study reports that the activation of mouse neuron within cortical slices using optogenetic stimulation would lead to secretion of a panel of neurotrophins including BDNF and neuroligin‐3. These neurotrophins in neuronal CM could act on gliomas and regulate their behaviors. Here, we first tried to reproduce these results using high KCl‐induced depolarization, which greatly enhances neurotrophin secretion from neurons (Nagappan *et al*., 2009). CM from depolarized neurons contained a large amount of BDNF, neuroligin‐3, CA11 and CA10, and CM promoted cell proliferation in two glioma cell lines. We also confirm that neuroligin‐3 is the major mediator, as immunodepletion of neuroligin‐3 abolished the effect of CM. But it is unclear whether any components within CM may antagonize neuroligin‐3‐mediated glioma growth from previous study. Here, we unexpectedly found that CA11 secreted by depolarized neurons could reduce neuronal activity‐dependent glioma growth. This suggests that neuronal CA11 could inhibit glioma growth in a paracrine manner. As CA11 is also expressed by glia themselves in normal brains, we further investigated the expression and function of glioma CA11. We found that CA11 reduction was associated with short survival in patients and CA11 knockdown promoted glioma aggression in various *in vitro* and *in vivo* assays. These results support that CA11 also inhibits glioma growth in an autocrine manner.

It is important to note that the high homology between CA10 and CA11 suggests that they may share conserved functions. Indeed, both CA10 and CA11 are extracellular binding partners for neurexins. Here, we show that both CA10 and CA11 were secreted by depolarized neurons, downregulated in gliomas, and associated with survival, and they have a similar inhibitory effect on glioma cell growth. Although we focused on CA11 in *in vitro* and *in vivo* assays, as our previous bioinformatics analysis identifies CA11 as part of a prognosis signature in gliomas, our study supports that CA10 and CA11 play a conserved and important role in gliomas.

Our study, together with other studies, supports a complex interaction between neurons and glioma cells. On one hand, neurons release various positive factors such as neuroglin‐3 and negative factors such as CA11/CA10 to modulate glioma behaviors. On the other hand, gliomas respond to each neuronal signal in different ways. Neuron‐derived neuroglin‐3 induces glioma neuroglin‐3 expression via a positive feedback mechanism, and we show here that unknown components in neuronal CM inhibit glioma CA11 expression, likely via the Akt signaling pathway. The paracrined CA11 from activated neurons and autocrined CA11 by gliomas coordinate to regulate glioma growth negatively, so that there is a fine‐tuned balance between the effects of neuroligin‐3 and CA11. The final balance of these factors in the neuron‐gliomas microenvironment may determine the readout as oncogenic or tumor‐suppressive. Thus, CA11/CA10 may represent a novel modulator which negatively regulates neuronal activity‐dependent glioma growth and inhibits aggressiveness of gliomas.

There are some limitations to our study. First, the binding partner of secreted CA11 on the surface of glioma cells is unknown. As CA11 is a ligand for neurexin, and glioma cells also express some types of neurotrophin receptors (Johnston *et al*., 2007; Wiesenhofer *et al*., 2000), it is possible that secreted CA11 binds to these receptors to modulate downstream signaling pathways. Secondly, although CM from depolarized neurons inhibited glioma CA11 expression, the component mediating this inhibitory effect is unknown. Our results suggest that neuroligin‐3 or CA11 within CM is unlikely to be such a mediator, and mass spectrometric analysis of CM might identify the potential mediators.

## 5 Conclusions

We report that depolarized neurons secrete CA11/CA10 to reduce glioma growth and release unknown factors to inhibit glioma CA11 expression via the Akt signaling pathway. CA11/CA10 expression is also reduced in clinical glioma samples, and low CA11/CA10 expression is associated with poor prognosis. In addition, CA11 knockdown promotes aggressive tumor behaviors in *in vitro* and *in vivo* assays. Our results suggest that CA11/CA10 negatively regulates neuronal activity‐dependent glioma growth and inhibits aggression of gliomas. Thus, CA11/CA10 is a potential therapeutic target for the treatment of gliomas.

## Conflict of interest

The authors declare no conflict of interest.

## Author contributions

BT, YL, YZ, and SL performed experiments, analyzed the data, and drafted the manuscript. PZ, XW, BL, ZJ, WZ, CX, JS, and LW repeated some results. WZ and SL designed the study and wrote the manuscript. All authors read and approved the final version of the manuscript.

## Supporting information


**Fig. S1**. Neuron secretes CA10 and CA10 reduces glioma cell growth.

**Fig. S2**. CA10 expression in databases.

**Fig. S3**. CA11 expression is not associated with survival in TCGA GBM dataset.

**Fig. S4**. Association of CA10 expression with survival in databases.

**Table S1**. Multivariate Cox proportional hazard model analysis.
