# Supplementary figures and images for: CA10 and CA11 negatively regulate neuronal activity‐dependent growth of gliomas
Source: Mol Oncol. 2019 Mar 20;13(5):1018–32. doi: 10.1002/1878-0261.12445 (PMC6487704; doi:10.1002/1878-0261.12445)

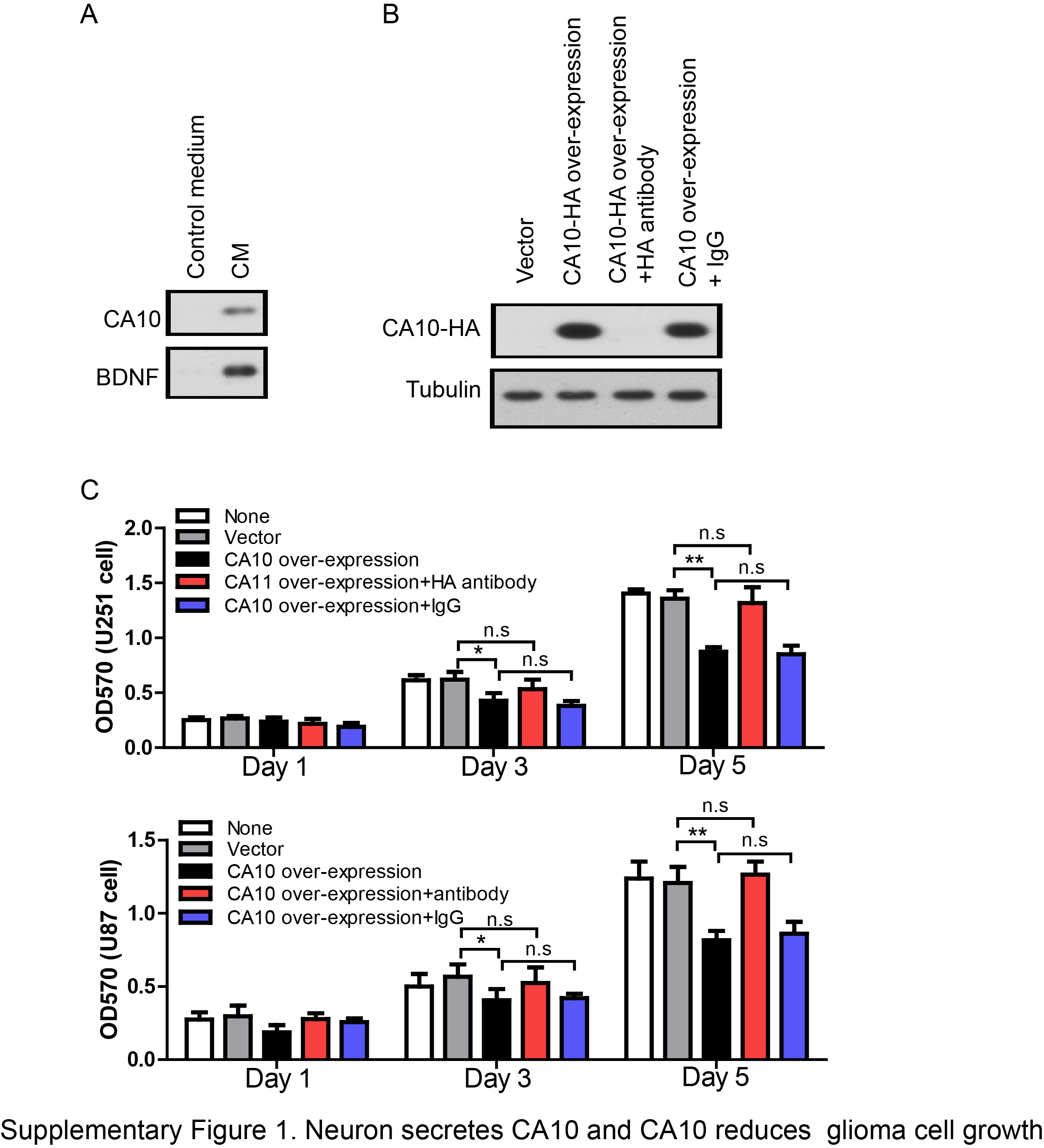

Supplement: Supplementary file 1 — Fig. S1. Neuron secretes CA10 and CA10 reduces glioma cell growth. [file MOL2-13-1018-s001.tif]

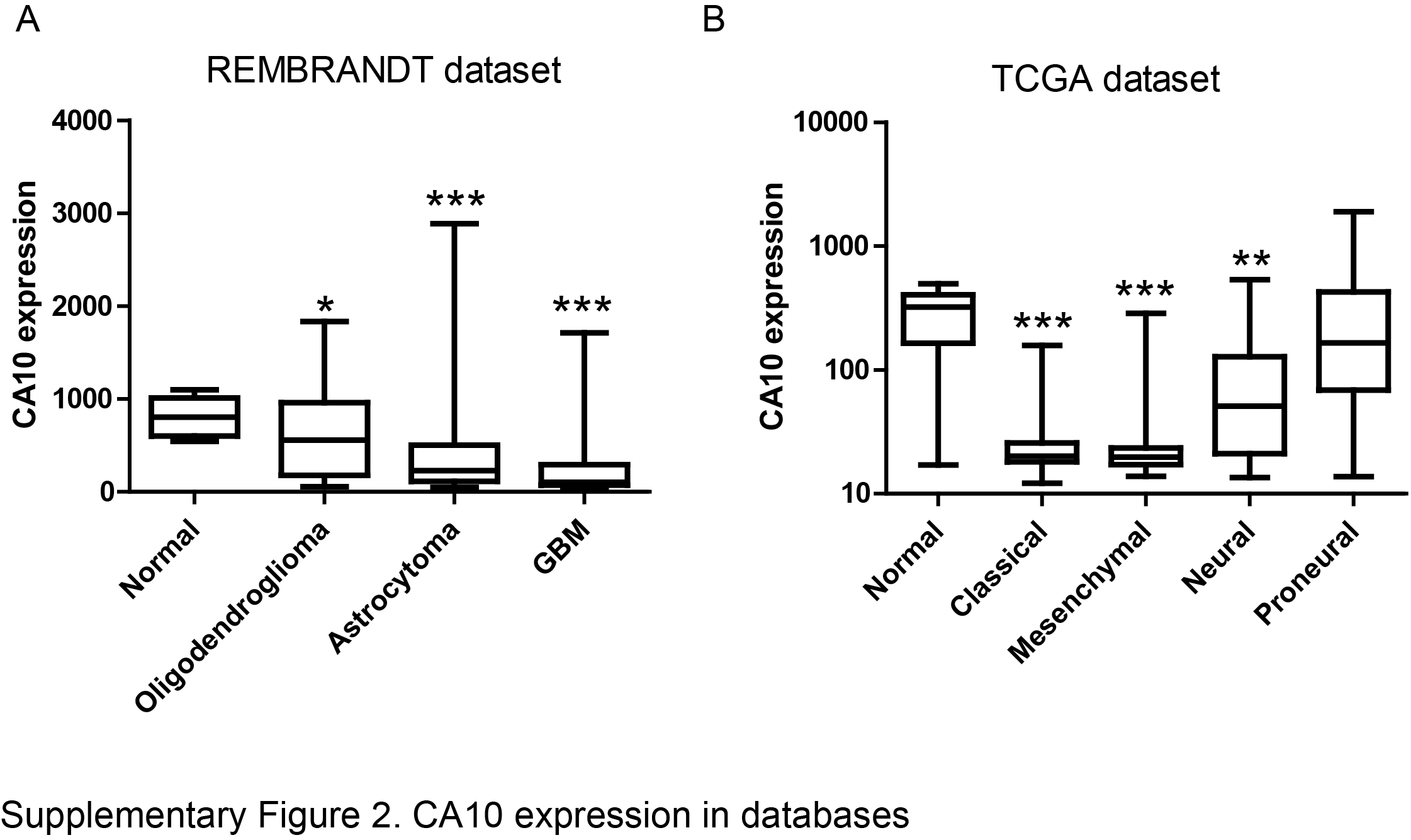

Supplement: Supplementary file 2 — Fig. S2. CA10 expression in databases. [file MOL2-13-1018-s002.tif]

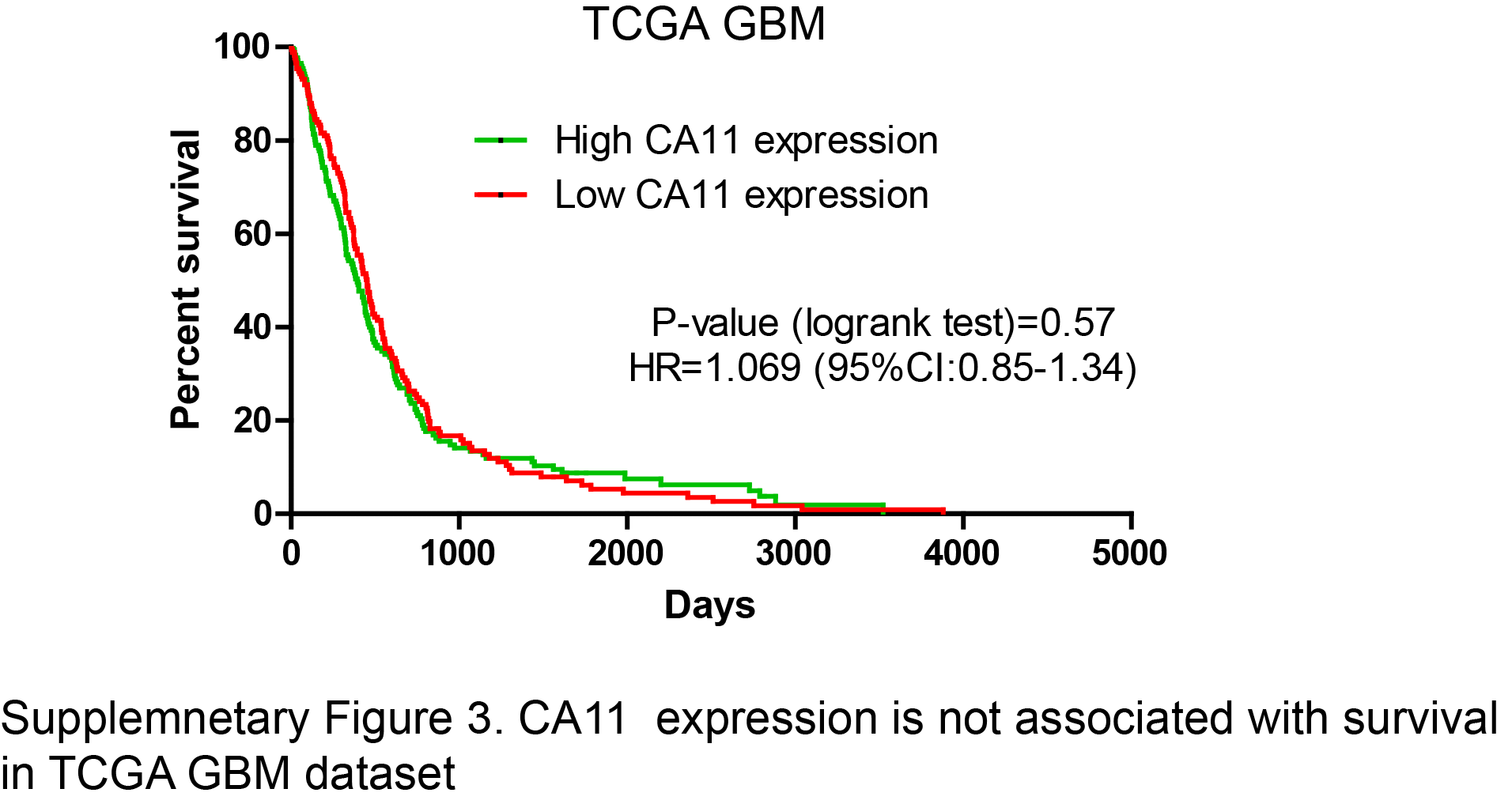

Supplement: Supplementary file 3 — Fig. S3. CA11 expression is not associated with survival in TCGA GBM dataset. [file MOL2-13-1018-s003.tif]

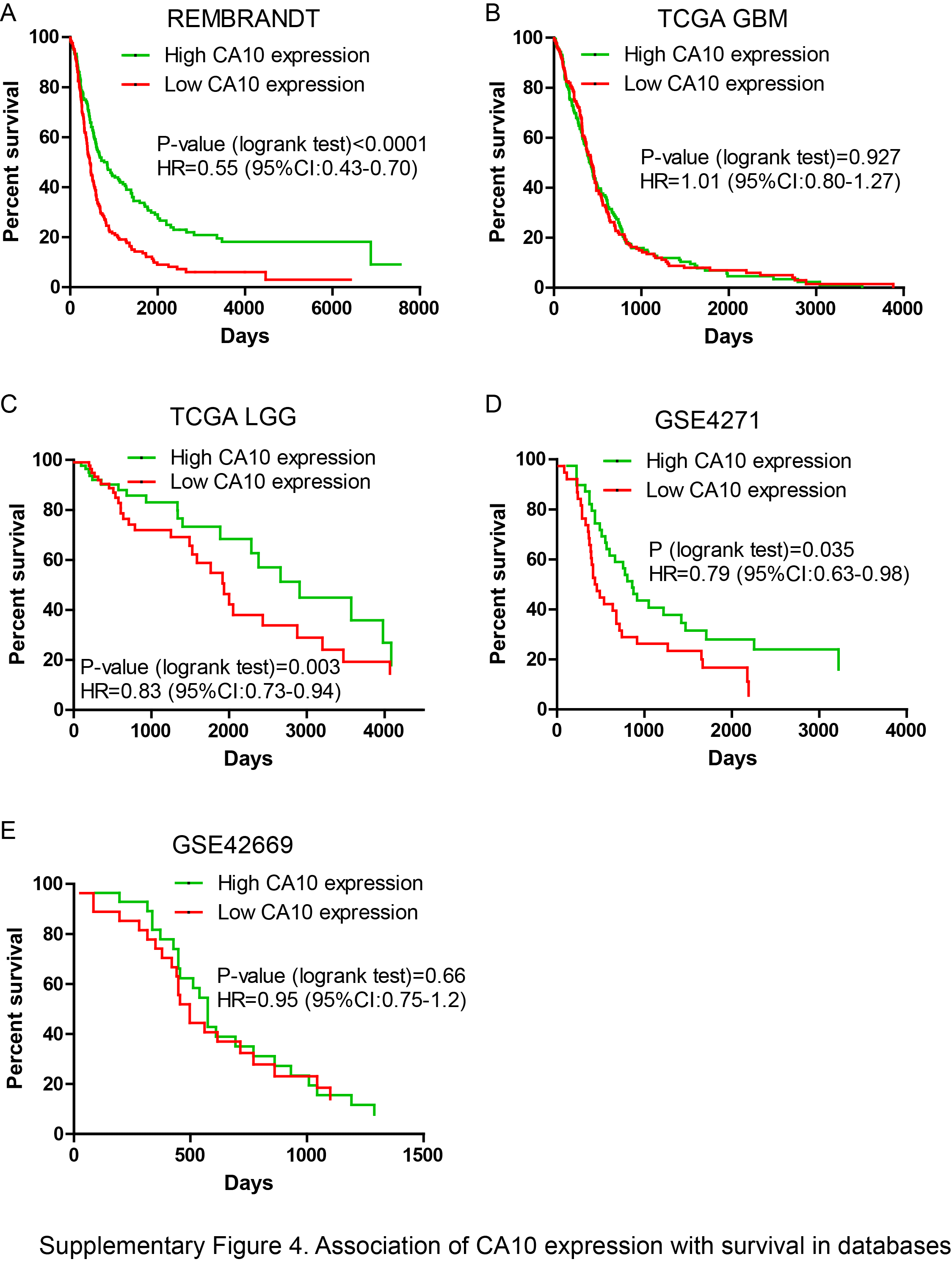

Supplement: Supplementary file 4 — Fig. S4. Association of CA10 expression with survival in databases. [file MOL2-13-1018-s004.tif]
