# Supplementary material for: CA10 and CA11 negatively regulate neuronal activity‐dependent growth of gliomas
Source: Mol Oncol. 2019 Mar 20;13(5):1018–32. doi: 10.1002/1878-0261.12445 (PMC6487704; doi:10.1002/1878-0261.12445)
Supplement: Supplementary file 5 — Table S1. Multivariate Cox proportional hazard model analysis. [file MOL2-13-1018-s005.doc]

Table S1. Multivariate Cox proportional hazard model analysis

| **Variables in the Equation** | | | | | | | | |
| --- | --- | --- | --- | --- | --- | --- | --- | --- |
|  | B | SE | Wald | df | Sig. | Exp(B) | 95.0% CI for Exp(B) | |
|  |  |  |  |  |  |  | Lower | Upper |
| CA11 Expression | .379 | .186 | 4.159 | 1 | .041 | 1.461 | 1.015 | 2.102 |
| Histopathological Grade | .408 | .187 | 4.729 | 1 | .030 | 1.503 | 1.041 | 2.171 |
