# Supplementary material for: CA10 and CA11 negatively regulate neuronal activity‐dependent growth of gliomas
Source: Mol Oncol. 2019 Mar 20;13(5):1018–32. doi: 10.1002/1878-0261.12445 (PMC6487704; doi:10.1002/1878-0261.12445)
Supplement: Supplementary file 6 [file MOL2-13-1018-s006.docx]

Supplementary Figure 1. Neuron secretes CA10 and CA10 reduces glioma cell growth

(A) Representative western blot showing CA10 and BDNF levels in the conditioned medium from control neurons and depolarized neurons. (B) Representative western blot showing CA10 levels in the conditioned medium from HEK293T cells over-expressing HA tagged human CA10 with or without immunodepletion of CA10. (C) MTT assay results showing the growth of U251 and U87 cells treated with indicated HEK293T conditioned medium at Day1, Day3 and Day5, respectively (n=5 biologically-independent replicates). For all, *P<0.05; **P<0.01 by one way ANOVA with Newman-Keuls post hoc test. The error bars in all the subfigures represent SD.

Supplementary Figure 2. CA10 expression in databases

CA10 expression data in (A) REMBRANDT glioma dataset and (B) TCGA GBM dataset. Data were presented as whiskers-box plots. For all, *P<0.05; **P<0.01; ***P<0.001 by Kruskal–Wallis test followed by post hoc Dunn’s multiple comparison test.

Supplementary Figure 3. CA11 expression is not associated with survival in TCGA GBM dataset

Kaplan-Meier survival curves of patients classified by CA11 expression in TCGA GBM dataset (HR=1.069, P=0.57).

Supplementary Figure 4. Association of CA10 expression with survival in databases

Kaplan-Meier survival curves of patients classified by CA10 expression in (A) REMBRANDT gliomas (HR=0.55, P<0.0001), (B) TCGA GBM (HR=1.01, P=0.927), (C) TCGA LGG (HR=0.83, P=0.003), (D) GSE4271 (HR=0.79, P=0.035) and (E) GSE42669 (HR=0.95, P=0.66).
